# Supplementary material for: Mechanistic insights into direct DNA and RNA strand transfer and dynamic protein exchange of SSB and RPA
Source: Nucleic Acids Res. 2025 Jul 2;53(12):gkaf642. doi: 10.1093/nar/gkaf642 (PMC12214019; doi:10.1093/nar/gkaf642)
Supplement: gkaf642_Supplemental_File [file gkaf642_supplemental_file.docx]

**SUPPLEMENTARY INFORMATION**

**Mechanistic insights into direct DNA and RNA strand transfer and dynamic protein exchange of SSB and RPA**

Tapas Paul^1,2^, I-Ren Lee^3^, Sushil Pangeni^1,4^, Fahad Rashid^5^, Olivia Yang^5^, Edwin Antony^6^, James M. Berger^5^, Sua Myong^1,2^ and Taekjip Ha^1,2,7, *^

^1^Program in Cellular and Molecular Medicine, Boston Children's Hospital, Boston, Massachusetts, USA.

^2^Department of Pediatrics, Harvard Medical School, Boston, Massachusetts, USA.

^3^Department of Chemistry, National Taiwan Normal University, Taipei 116, Taiwan.

^4^T. C. Jenkins Department of Biophysics, Johns Hopkins University, Baltimore, Maryland, USA.

^5^Department of Biophysics and Biophysical Chemistry, Johns Hopkins University School of Medicine, Baltimore, Maryland, USA.

^6^Department of Biochemistry and Molecular Biology, St. Louis University School of Medicine, St. Louis, Missouri, USA.

^7^Howard Hughes Medical Institute, Boston, Massachusetts, USA.

*Correspondence should be addressed to T.H. (taekjip@childnres.harvard.edu).

Supplementary information includes:

Supplementary Table S1-S2

Supplementary Figures, S1-S7

**Supplementary Table 1.** DNA Oligonucleotides (5’ to 3’) used in this experiment.

| 18mer-poly(dT)_Cy3 | TGG CGA CGG CAG CGA GGC (T)_n_-/Cy3/ (n = 40, 70) |
| --- | --- |
| Cy5-18mer-Bio | /Cy5/-GCC TCG CTG CCG TCG CCA-/Bio/ |
| Unlabeled Poly(dT) | (dT)_n_ (n = 40, 60) |
| Poly(dT)_Cy3 | (dT)_40_-Cy3 |
| 18mer-poly(dT) | TGG CGA CGG CAG CGA GGC (T)_n_ (n = 40, 70) |
| 18mer-poly-uracil _Amine | /Amine/-(rU)_50_ rGrCrC rUrCrG rCrUrG rCrCrG rUrCrG rCrCrA |
| Bio -18mer-Cy5 | /Bio/-rUrGrG rCrGrA rCrGrG rCrArG rCrGrA rGrGrC-/Cy5/ |
| Unlabeled Poly-uracil | (rU)_50_ |
| Mixed seq_Cy3 | TGGCGACGGCAGCGAGGCTAAATTAATACGACTCACTATAGGGAGACCACAAGG/T-Cy3/GAGGAGTAGGAATCCGTATCTATCAGCTCCAGG |
| Comp_mixed seq | TAAATTAATACGACTCACTATAGGGAGACCACAAGGTGAGGAGTAGGAATCCGTATCTATCAGCTCCAGG |

**Supplementary Table 2.** Summary of exchange rate constants measured in this study.

| **Transfer or Exchange conditions** | **Exchange rate (M^-1^ s^-1^)** |
| --- | --- |
|  |  |
| dT_70_-SSB bound transfer to dT_60_ | 6.1 ± 0.1 x 10^4^ |
| dT_40_-SSB bound transfer to dT_40_ | 9.8 ± 0.2 x 10^4^ |
|  |  |
| dT_40_-RPA bound transfer to dT_40_ | 8.6 ± 0.3 x 10^4^ |
| dT_70_-RPA bound transfer to dT_60_ | 9.1 ± 0.4 x 10^4^ |
|  |  |
| dT_40_-hRPA bound transfer to dT_40_ | 8.3 ± 0.6 x 10^4^ |
|  |  |
| dT_70_-SSB bound exchange with SSB | 4.8 (± 0.8) 10^5^ |
| dT_40_-SSB bound exchange with SSB | 3.8 (± 0.3) 10^5^ |
|  |  |
| dT_40_-RPA bound exchange with RPA | 2.6 ± 0.6 x 10^5^ |
| dT_40_-RPA bound exchange with RPA | 2.7 ± 0.2 x 10^5^ |
|  |  |
| U_50_-SSB bound transfer to U_50_ | 2.1 (± 0.2) x 10^5^ |
| U_50_-RPA bound transfer to U_50_ | 4.3 (± 0.4) x 10^5^ |

**Supplementary Figure 1**


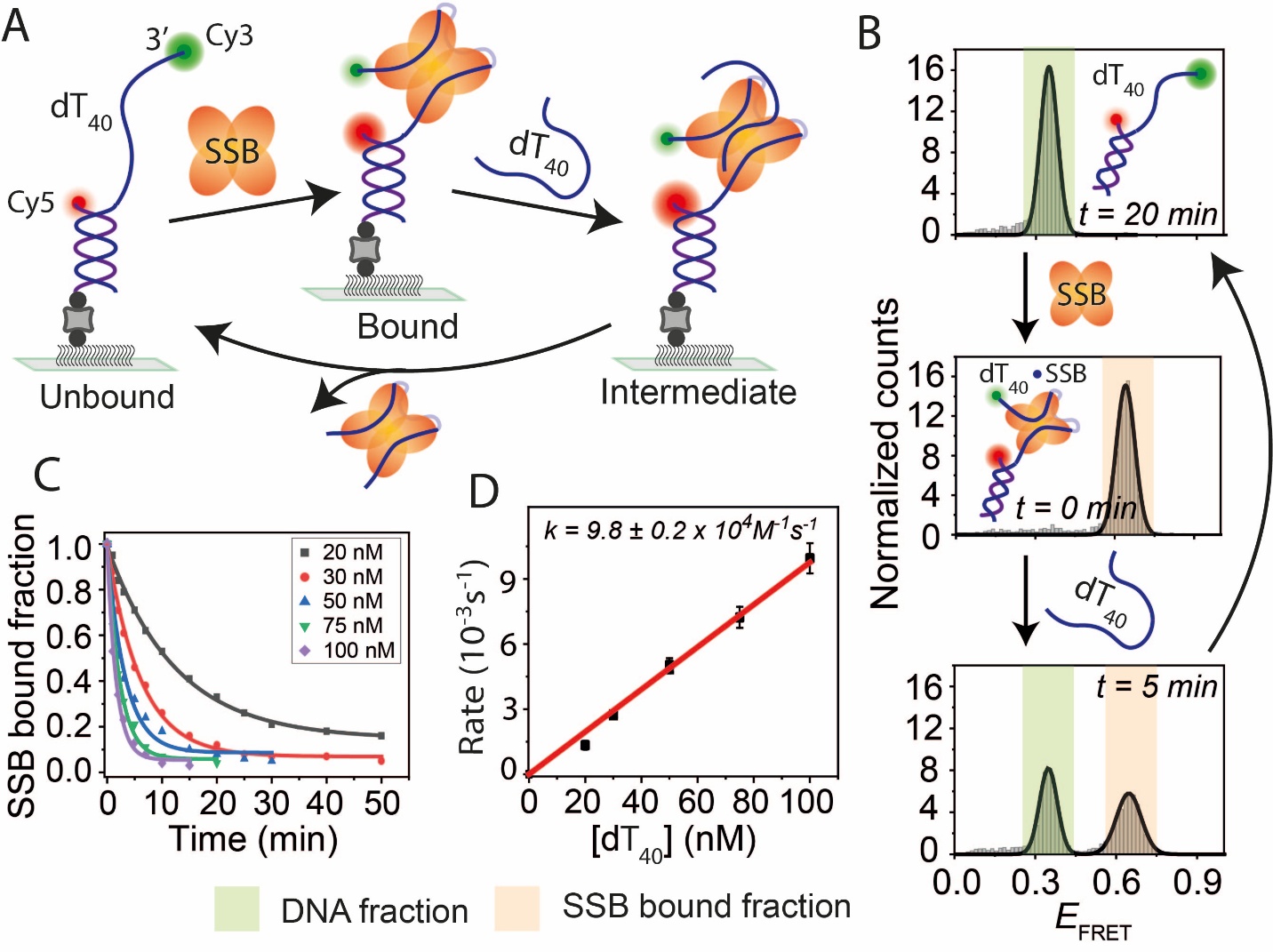


**Supplementary Figure 1:** SSB binding and direct transfer kinetics on dT_40_. (A) Schematic of smFRET constructs showing a partial DNA duplex with a 40-nt poly(dT) overhang (dT_40_). SSB binds to dT_40_, followed by strand transfer to competing ssDNA (dT_40_). The binding and transfer reactions were conducted in a buffer containing 100 mM NaCl. (B) FRET histograms of dT_40_ before (top) and after SSB binding (middle), with the bottom histogram showing FRET changes during SSB transfer at indicated times. Time, t=20 min (top histogram) represents the complete strand transfer of bound SSB. (C) Single-exponential fitting of the SSB-bound fraction at different dT_40_ concentrations. (D) Linear fit of SSB transfer rates at varying dT_40_ concentrations.

**Supplementary Figure 2**


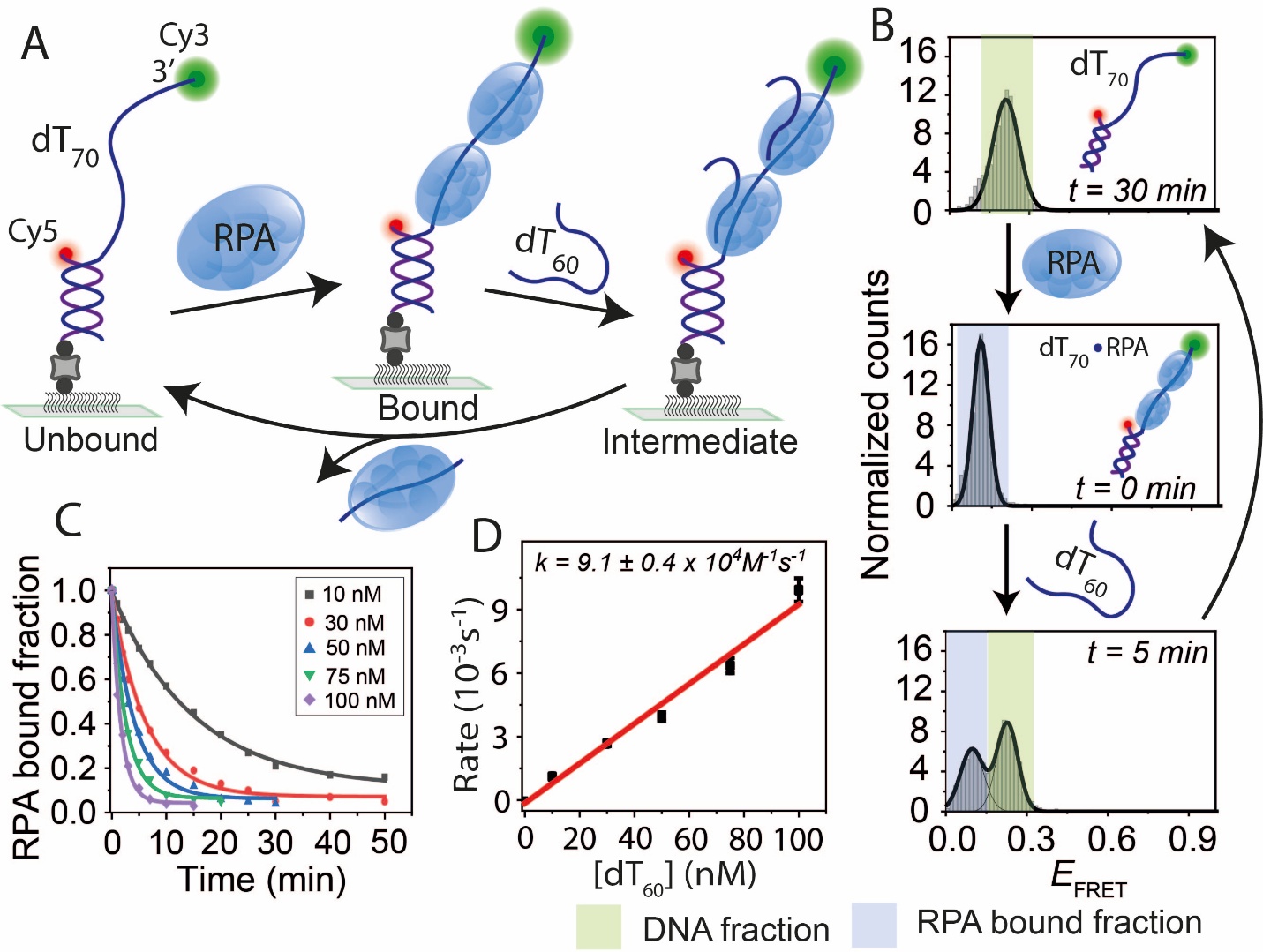


**Supplementary Figure 2:** RPA binding and direct transfer kinetics on dT_70_. (A) Schematic of smFRET constructs showing a partial DNA duplex with a 70-nt poly(dT) overhang (dT_70_). Budding yeast RPA binds to dT_70_, followed by strand transfer to competing ssDNA (dT_60_). The binding and transfer reactions were conducted in a buffer containing 100 mM NaCl. (B) FRET histograms of dT_70_ before (top) and after RPA binding (middle), with the bottom histogram showing FRET changes during RPA transfer at indicated times. Time, t=30 min (top histogram) represents the complete strand transfer of bound RPA. (C) Single-exponential fitting of the RPA-bound fraction at different dT_60_ concentrations. (D) Linear fit of RPA transfer rates at varying dT_60_ concentrations.

**Supplementary Figure 3**


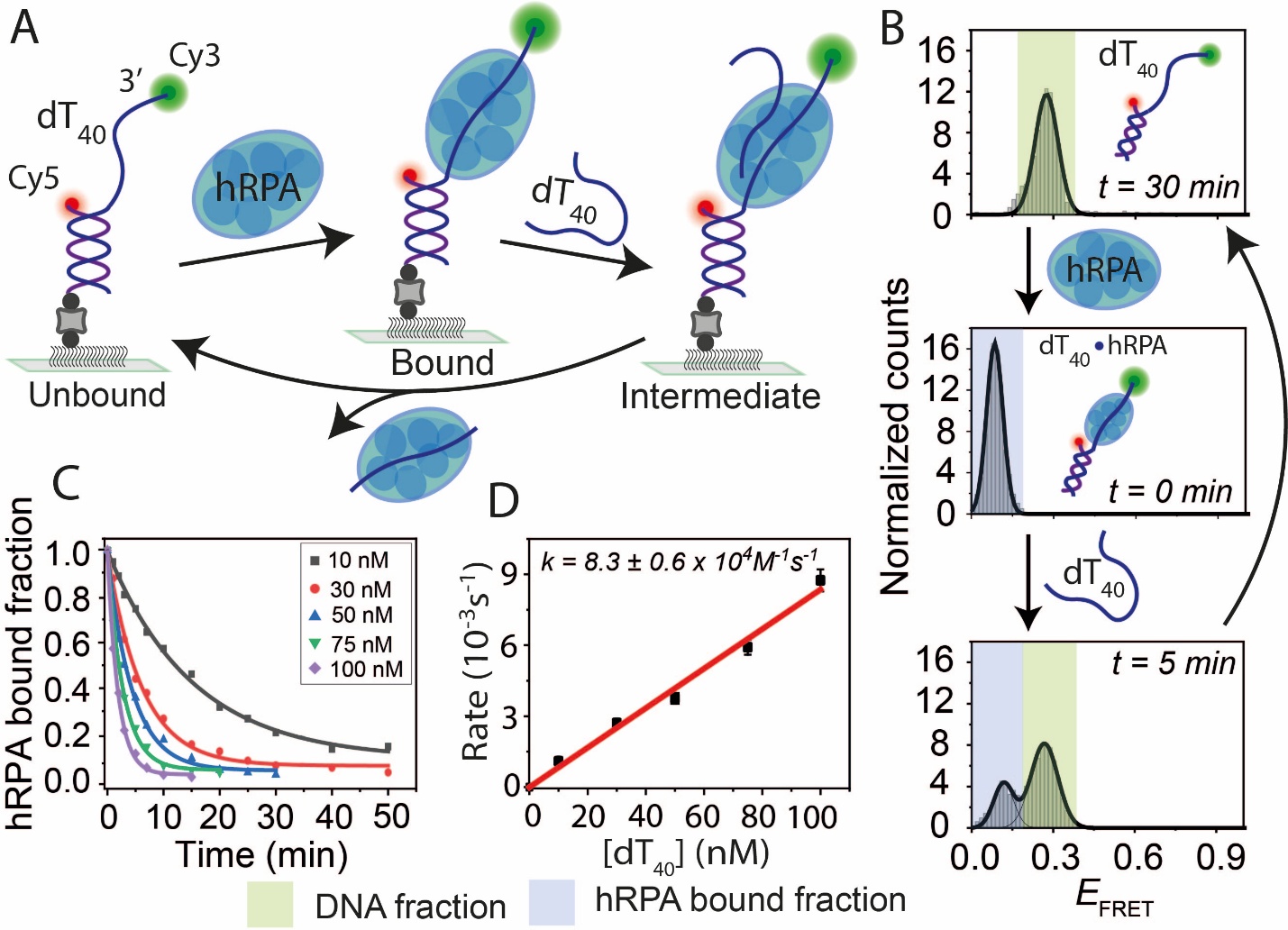


**Supplementary Figure 3:** Human RPA (hRPA) binding and transfer on dT_40_. (A) Schematic of smFRET constructs showing a partial DNA duplex with a 40-nt poly(dT) overhang (dT_40_). Human RPA (hRPA) binds to dT_40_, followed by strand transfer to competing ssDNA (dT_40_). The binding and transfer reactions were conducted in a buffer containing 100 mM NaCl. (B) FRET histograms of dT_40_ before (top) and after hRPA binding (middle), with the bottom histogram showing FRET changes during hRPA transfer at indicated times. Time, t=30 min (top histogram) represents the complete strand transfer of bound RPA. (C) Single-exponential fitting of the hRPA-bound fraction at different dT_40_ concentrations. (D) Linear fit of hRPA transfer rates at varying dT_40_ concentrations.

**Supplementary Figure 4**


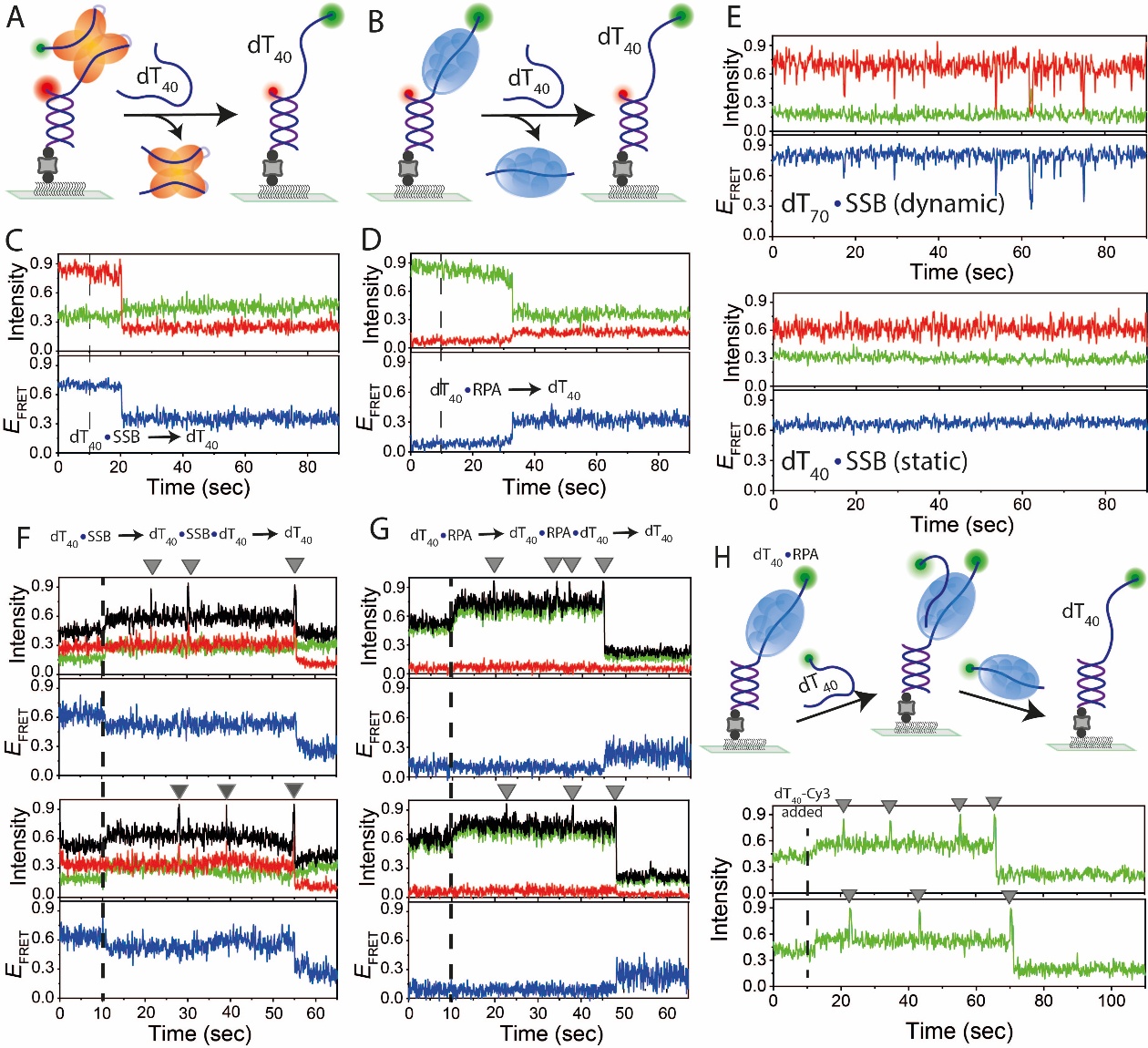


**Supplementary Figure 4:** Real-time smFRET traces of SSB and RPA transfer. (A, B) Schematic smFRET model of dT_40_ bound with SSB (A) or RPA (B) transfer to the competing dT_40_ ssDNA and regenerate the tethered DNA. (C, D) Representative real-time smFRET traces showing one-step FRET transitions during SSB (C) and RPA (D) transfer to competing ssDNA (100 nM). Dashed lines indicate the addition of competing ssDNA. (E) Representative real-time smFRET traces showing the dynamics of SSB bound to dT_70_ (top, dynamic binding) and dT_40_ (bottom, static binding). (F, G) Real-time smFRET traces showing SSB (F) and RPA (G) transfer events. Dash line indicate Cy3-ssDNA flow; spikes represent transfer attempts (gray triangles), and the final spike referred to successful transfers with intensity and FRET efficiency changes. The background intensity increases after addition of Cy3-labeled ssDNA. Black, red, green, and blue lines indicate total intensity, Cy5 intensity, Cy3 intensity, and FRET efficiency, respectively. (H) Schematic model of Cy3-labeled dT_40_ bound with RPA transfer to the competing dT_40_ with Cy3-labeled ssDNA and regenerate the tethered DNA. Time traces showing RPA transfer events. Dash line indicate Cy3-ssDNA flow; spikes represent transfer attempts (gray triangles), and the final spike referred to successful transfers with intensity changes. The background intensity increases after addition of Cy3-labeled ssDNA (~10 sec).

**Supplementary Figure 5**


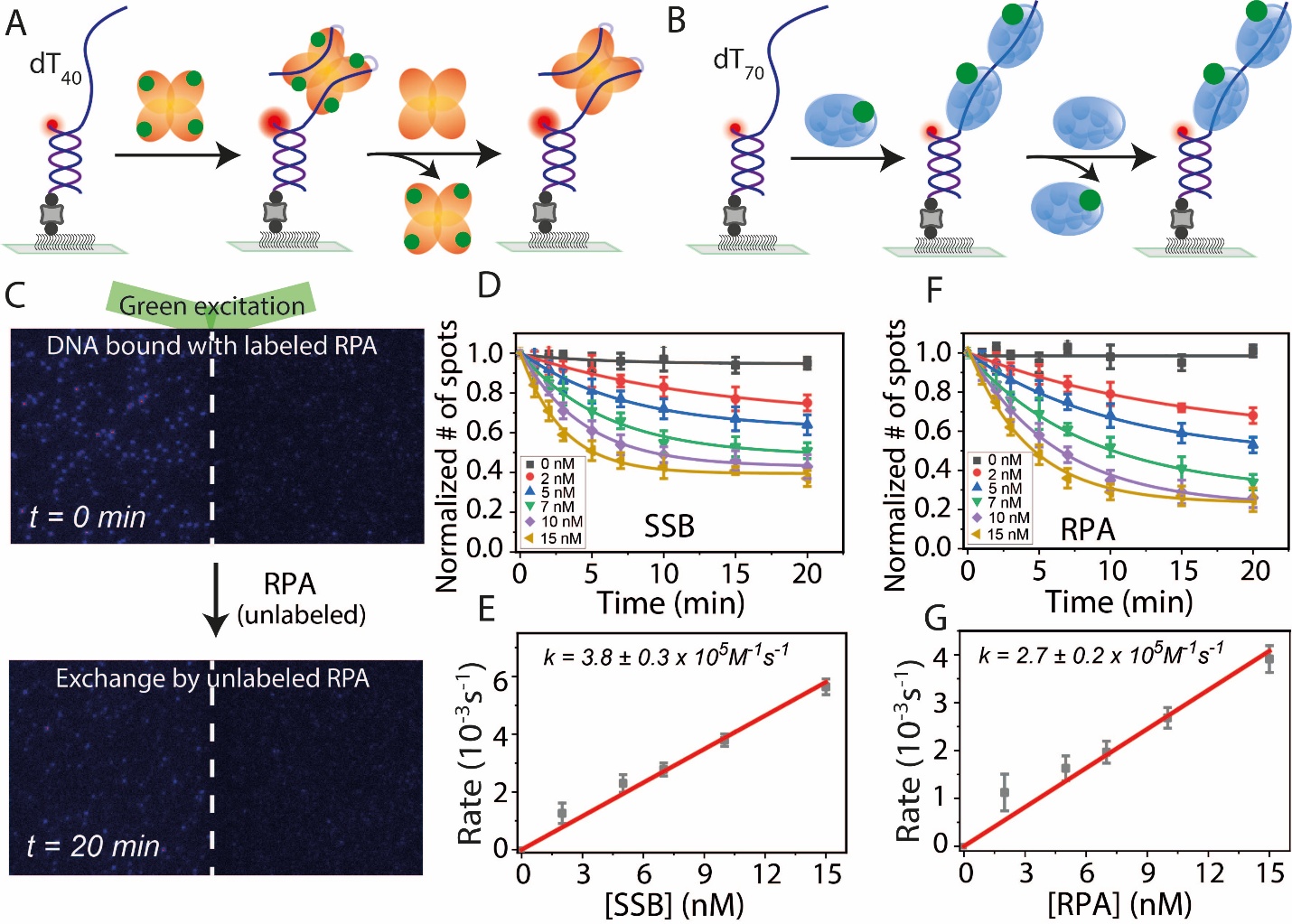


**Supplementary Figure 5:** Protein-protein exchange dynamics of SSB and RPA. (A, B) Schematic representation of SSB (A) and RPA (B) protein-protein exchange, where labeled proteins bound to DNA are replaced by unlabeled proteins. The binding and exchange reactions were conducted in a buffer containing 100 mM NaCl. (C) Representative fields of view showing labeled RPA at t=0 min (top) and after exchange with unlabeled protein at t=20 min (bottom) under green laser excitation. (D, F) Single-exponential fits of labeled protein disappearance at different unlabeled protein concentrations for SSB (D) and RPA (F). (E, G) Linear fits of exchange rates at varying protein concentrations for SSB (E) and RPA (G).

**Supplementary Figure 6**


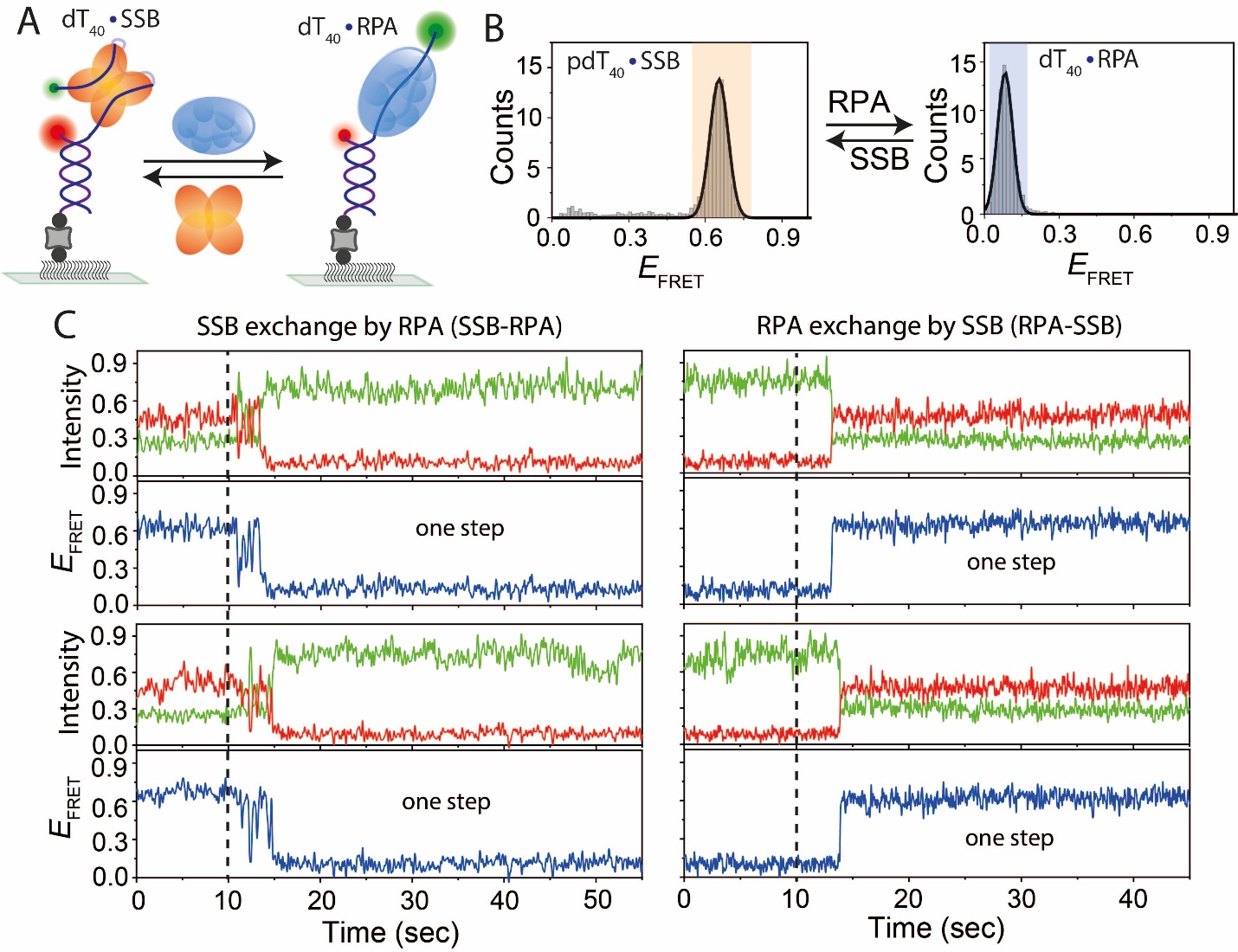


**Supplementary Figure 6:** Hetero-protein exchange dynamics between SSB and RPA. (A) Schematic smFRET model showing hetero-protein exchange between SSB and RPA. The binding and exchange reactions were conducted in a buffer containing 100 mM NaCl. (B) FRET histograms of dT_40_ showing SSB and RPA binding and their exchange. (C) Real-time smFRET traces showing SSB-to-RPA and RPA-to-SSB exchanges on dT_40_. Distinct FRET transitions indicate protein exchange events, with dashed lines marking protein addition.

**Supplementary Figure 7**


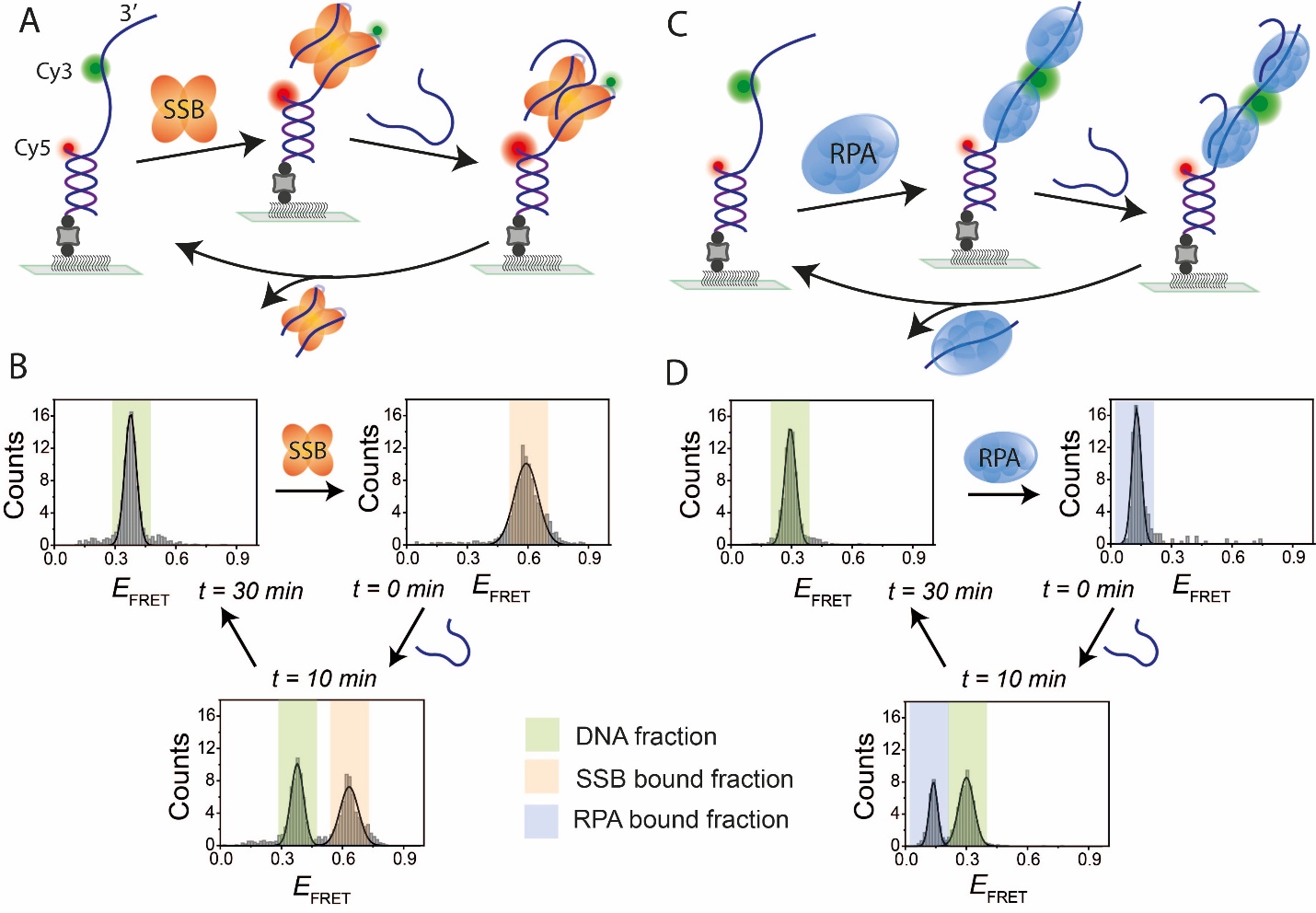


**Supplementary Figure 7:** SSB and RPA binding and strand transfer on mixed-sequence ssDNA. (A, C) Schematic of smFRET constructs showing a partial duplex DNA with a 70-nt hetero-base overhang. SSB (A) and RPA (C) bind to the 70-nt mixed-sequence overhang, followed by strand transfer to a competing ssDNA strand of identical sequence. (B, D) FRET histograms showing the DNA-only state (left), protein-bound state (right), and time-dependent strand transfer (bottom) for SSB (B) and RPA (D). SSB experiments were performed in buffer containing 300 mM NaCl, and RPA experiments in buffer with 100 mM NaCl. The hetero-base sequences used are listed in Supplementary Table 1.
